# Dispersive liquid-liquid microextraction for the isolation and HPLC-DAD determination of three major capsaicinoids in Capsicum annuum L.

**DOI:** 10.3906/kim-2009-55

**Published:** 2021-04-28

**Authors:** Jude CALEB, Usama ALSHANA, Azmi HANOĞLU, İhsan ÇALIŞ

**Affiliations:** 1 Department of Analytical Chemistry, Faculty of Pharmacy, Near East University, Lefkoşa, TRNC, Mersin 10 Turkey; 2 Department of Pharmaceutical Botany, Faculty of Pharmacy, Near East University, Lefkoşa, TRNC, Mersin 10 Turkey; 3 Department of Pharmacognosy, Faculty of Pharmacy, Near East University, Lefkoşa, TRNC, Mersin 10 Turkey

**Keywords:** Capsaicin, *Capsicum annuum*L., dihydrocapsaicin, dispersive liquid-liquid microextraction, nordihydrocapsaicin

## Abstract

Dispersive liquid-liquid microextraction (DLLME) was combined with high-performance liquid chromatography-diode-array detector (HPLC-DAD) for the extraction and quantitation of three major capsaicinoids (i.e. capsaicin, dihydrocapsaicin and nordihydrocapsaicin) from pepper (*Capsicum annuum* L.). Chloroform (extraction solvent, 100 μL), acetonitrile (disperser solvent, 1250 μL) and 30 s extraction time were found optimum. The analytes were back-extracted into 300 μL of 50 mM sodium hydroxide/ methanol, 45/55% (v/v), within 15 s before being injected into the instrument. Enrichment factors ranged from 3.3 to 14.7 and limits of detection from 5.0 to 15.0 µg g^-1^. Coefficients of determination (R^2^) and %RSD were higher than 0.9962 and lower than 7.5%, respectively. The proposed method was efficiently applied for the extraction and quantitation of the three capsaicinoids in six cultivars of *Capsicum annuum* L. with percentage relative recoveries in the range of 92.0%–108.0%. DLLME was also scaled up for the isolation of the three major capsaicinoids providing purity greater than 98.0% as confirmed by liquid chromatography-mass spectrometry (LC-MS) and nuclear magnetic resonance (NMR) analysis, which significantly reduced the extraction time and organic solvent consumption.

## 1. Introduction

Peppers (*Capsicum annuum* L.), originating in the Americas, are popularly known for their spicy and pungent taste, which are caused by a group of molecules known as capsaicinoids from the genus capsicum, making them a popular spice in foods around the world [1]. Although over twenty capsaicinoids have been identified in various species of pepper [2], only two of them are responsible for up to 90% of the pungency of pepper, namely, capsaicin (CAP) and dihydrocapsaicin (DHC) [3]. The other capsaicinoids include nordihydrocapsaicin (NDHC), homodihydrocapsaicin and homocapsaicin [4]. The level of pungency depends on the species and cultivars, in which the concentration of capsaicinoids is influenced by certain variables such as the type of soil, climate and other growth conditions [5]. Capsaicinoids, especially CAP, are primarily used as coloring and/or flavoring agents in the food and pharmaceutical industry. Studies revealed that CAP can suppress carcinogenesis in the breast, prostrate, colon, lungs and human bladder [6]. It is used for topical applications in analgesic therapy for some neuropathic and osteoarthritic pain states [7] and has recently, been found to alleviate rheumatoid arthritis [8]. In addition, capsaicinoids have been reported to possess antimicrobial effects against disease-causing bacterial and aquatic microorganisms that cover underwater areas of ships [9]. Despite the important pharmacological, clinical and industrial uses of CAP, high doses (above 100 mg per body weight) administered for a long time might cause peptic ulcers, increase the chances of developing liver, duodenal, stomach and prostate cancer together with the enhancement of breast cancer metastasis [10].

The concentration of CAP in pepper is one of the essential factors that define its competitive value [11] and thus the need for rapid, high throughput analysis at a reduced cost has placed a high demand for developing efficient analytical methods for food analysis [12]. The short analysis time and reliability of high-performance liquid chromatography (HPLC) made it the favored technique for separating capsaicinoids. Recently, several HPLC methods with ultraviolet (UV) [13], fluorescence [14], electrochemical [15] and mass spectrometry (MS) [16] detectors have been reported for this purpose. Despite the substantial technological achievements made in HPLC instrumentation, it is still challenging to achieve rapid, highly efficient and highly selective separation of food matrices without an efficient sample preparation step [17] owing to the high complexity of such samples. The drawbacks of conventional sample preparation techniques such as liquid-liquid extraction (LLE) and solid-phase extraction (SPE) are well known and documented in the literature, some worth mentioning are the tedious procedures and large consumption of toxic organic solvents involved in LLE, which are harmful to the researcher, living organisms and the environment. SPE uses smaller volumes of organic solvents, but it is still considered significant. In addition, SPE cartridges are cost-prohibitive and generate chemical waste [18].

The emphasis has recently been diverted towards the design of reliable miniaturized sample preparation techniques. Dispersive liquid-liquid microextraction (DLLME) [19] has achieved broad acceptance and recognition in food analysis [20–22] due to its rapidness, environmental friendliness, high extraction efficiency and affordability. In DLLME, the maximum extraction efficiency is maintained almost instantly thanks to the extremely large surface area of the extraction solvent available for the analyte. Although gas chromatography (GC) was the first instrument to be used with DLLME [19], in which the extract can be directly injected due to the compatibility of the final extract with the instrument, HPLC is so far, the most frequently used one after DLLME [23]. Evaporating the solvent to dryness and reconstituting in the mobile phase or back-extraction into a compatible solvent are common sample introduction methods of the final extract into reversed-phase HPLC after DLLME [24]. Back-extraction of ionizable compounds is a faster choice that can also provide additional selectivity, improved sample clean up and better regulation of the ionic strength in the final extract.

The objective of this research was to design a fast, robust and efficient extraction method using DLLME followed by back-extraction for preconcentration and determination of the three major capsaicinoids (i.e. NDHC, CAP and DHC) from different cultivars of *Capsicum annuum* L. using HPLC-diode-array detector (DAD). To date, this is the first study on DLLME of capsaicinoids from pepper samples. Influential experimental parameters on the extraction and back-extraction efficiencies were optimized. This was also the first attempt to scale up DLLME for the isolation of the three major capsaicinoids from *Capsicum annuum* L.

## 2. Experimental

### 2.1. Chemicals and reagents

**Figure 1:**
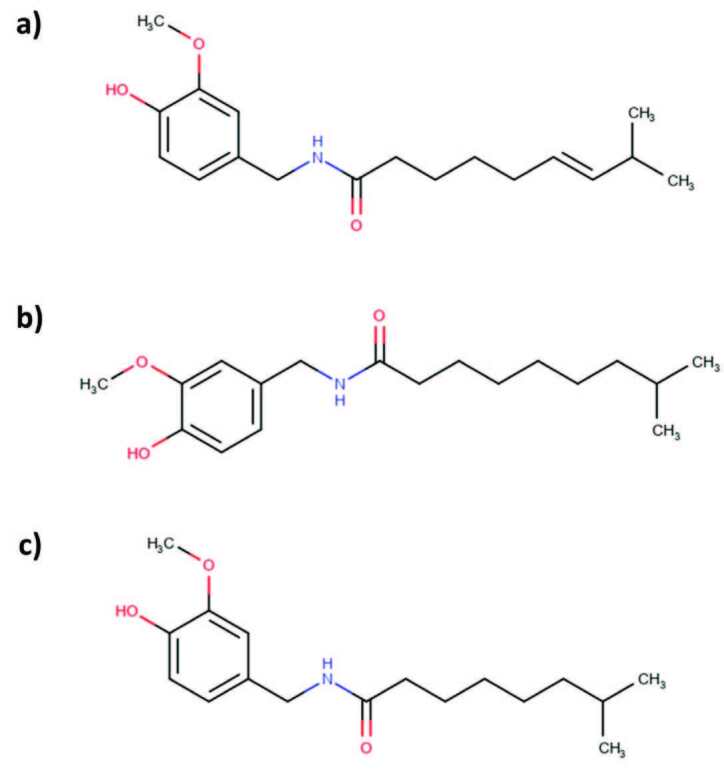
Chemical structures of the analytes: (a) capsaicin, (b) dihydrocapsaicin, and (c) nordihydrocapsaicin.

Analytical grade standards of CAP (logP = 3.75), DHC (logP = 4.11) and NDHC (logP = 3.67) were isolated on a preparative scale with purity above 98% from *Capsicum annuum* L. in our pharmacognosy laboratory, as confirmed by LC-MS and NMR analysis (supplementary material). The chemical structures of the three analytes are shown in Figure 1. HPLC-grade methanol, acetonitrile, tetrahydrofuran, chloroform, dichloromethane, sodium chloride, sodium hydroxide (NaOH) and ethyl acetate were acquired from Sigma-Aldrich (Darmstadt, Germany). Acetic acid, n-hexane and toluene were obtained from Honeywell Riedel-de Haën (Seelze, Germany). Silica gel was obtained from Merck (Søborg, Denmark). All reagents used were of analytical-grade. Deionized (DI) water (18.2 MΩ.cm), treated with Purelab Ultra Analytic (ELGA LabWater, Leicestershire, UK), was used for the preparation of aqueous solutions. MarvinSketch (Version 20.11.0, ChemAxon, Boston, MA, USA) was used for logP and p*K*a calculations.

### 2.2. Standard solutions of capsaicinoids 

Individual standards of the three capsaicinoids were prepared at 1000 µg mL^-1^ in methanol and stored at –4 °C. Mixed standard solutions were freshly prepared by diluting the stock solution with 50 mM sodium hydroxide/ methanol (45/55%, v/v), hereafter, referred to as the back-extraction solution (BES). Mobile phases and aqueous solutions were filtered using a vacuum solvent filtration system through 0.20 µm regenerated cellulose-membrane filters (Isolab, Eschau, Germany), whereas, samples were filtered through 0.22 µm nylon syringe filters (Microlab Scientific, Shanghai, China).

### 2.3. Instrumentation

An HPLC system (1200 series, Agilent Technologies, Santa Clara, CA, USA) with an online degasser, a quaternary pump, an autosampler, a column oven and DAD set at a wavelength of 280 nm was used for separations. Maximum absorption wavelength was selected using the ‘Isoabsorbance’ and ‘3D’ plot functions available in the Agilent ChemStation for LC 3D systems (Rev. B.03.01) software. A reversed-phase column (i.e. Agilent Zorbax SB-Aq 150 mm × 4.6 mm × 5 µm) was used to separate the analytes using an optimum isocratic mobile phase composition of 0.5% acetic acid/ methanol (55/45%, v/v) at a flow rate of 1.2 mL min-1 and 22 °C. A medium-pressure liquid chromatograph (MPLC) (BÜCHI Labortechnik AG, Flawil, Switzerland), equipped with a binary pump, a fraction collector and a reversed-phase column (i.e. LiChroprep RP-18 460 mm × 26 mm × 25–40 µm) (Millipore, Dachstein, France), was used for isolation of the analytes. LC-MS with electrospray ionization (ESI) was used for mass analysis (Waters Alliance HPLC and ZQ micromass, Waters Corp., Milford, MA, USA). For structural elucidation, spectral analysis was performed using a Varian Mercury Fourier-transform NMR (FT-NMR) spectroscopy (^1^H 400 MHz, ^13^C 100 MHz) (Agilent Technologies). Deuterated dimethyl sulfoxide (DMSO-d_6_) and chloroform (CDCl_3_) (Sigma-Aldrich, Darmstadt, Germany) were used as the solvents. Tetramethylsilane (TMS, Sigma-Aldrich, Darmstadt,Germany) was used as the internal standard. 

### 2.4. Sample preparation

Fresh cultivars of *Capsicum annuum* L. and green pepper pickles were collected from local markets in Nicosia, TRNC. The samples were washed with plenty of DI water, left to dry at room temperature, and cut into small pieces with a stainless steel knife. Then, they were dried overnight in the oven at 60 °C. Dry samples were powdered using a blender with stainless steel blades and stored in well-sealed glass bottles in a dark place till analysis. An initial solid-liquid extraction (SLE) was carried out by weighing 1.00 ± 0.01 g of the sample and extracting it with 50.0 mL of 50/50 (%, v/v) acetonitrile/ water through ultrasonication for 30 min at 25 °C. The mixture was filtered through a cotton wool and a 0.45 µL syringe filter and the filtrate was transferred into a 50 mL volumetric flask and made up to the mark with 50/50 (%, v/v) acetonitrile/water (referred to hereafter as the sample solution). A portion of 5.0 mL of the sample solution was mixed with 2.0 mL of saturated sodium chloride solution. The mixture was vortexed for 30 s and centrifuged for 3 min at 6000 rpm to induce salting-out extraction (SOE). Out of the 1.2 mL of acetonitrile that salted out, 1.0 mL was used in DLLME.

### 2.5. Dispersive liquid-liquid microextraction

One milliliter of acetonitrile, resulting from SOE, was completed to 10.0 mL with DI water into a 15 mL centrifuge tube after adding an extra volume of 250 μL of acetonitrile, which acted as the disperser solvent in DLLME. Upon the addition of chloroform and acetic acid (100 µL, each), the mixture was vortex mixed for 30 s and centrifuged (3 min at 6000 rpm). Chloroform, which settled to the bottom, was transferred completely into a snaplock microtube and the analytes were back-extracted into 300 µL of BES through vortexing for 15 s and centrifugation (3 min, 6000 rpm). A portion of the aqueous extract (i.e. 5 µL) from the upper layer was injected into HPLC-DAD for analysis.

## 3. Results and discussion

### 3.1. Optimization of extraction conditions

#### 3.1.1. Type and volume of the extraction solvent

**Figure 2:**
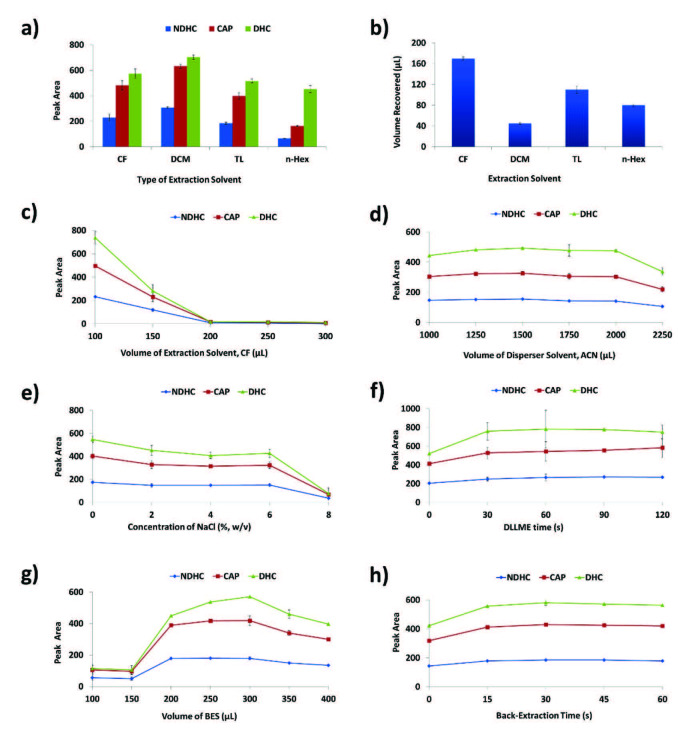
Optimization of DLLME parameters: (a) type of extraction solvent, (b) recovered volume of extraction solvent, (c) volume of extraction solvent, (d) volume of disperser solvent, (e) concentration of sodium chloride, (f) DLLME time, (g) volume of backextraction solvent (BES), and (h) back-extraction time. CF: chloroform; DCM: dichloromethane; n-Hex: n-hexane; TL: toluene.

Selecting a suitable extraction solvent for DLLME is based on its ability to extract the analyte(s) from the sample solution and its immiscibility with the aqueous phase [25]. In conventional DLLME, extraction solvents denser than water, such as chloroform, dichloromethane and tetrachloromethane, are commonly used [19]. However, the use of less dense solvents has also been proposed, which include toluene, n-hexane, 1-undecanol and 2-dodecanol [26]. Chloroform, dichloromethane, toluene and n-hexane were tested as the extraction solvents considering extractability and solvent recovery. Although dichloromethane gave the highest extraction efficiency (Figure 2a), the recovered volume of chloroform (Figure 2b) was much higher (i.e. 170 ± 5 µL), making it an optimum extraction solvent to proceed with. Volume of the selected solvent can significantly influence the extraction efficiency [21]. The effect of chloroform volume was monitored starting from 100 to 300 μL within 50 μL intervals. Peak areas rapidly decreased as the volume of chloroform was increased due to dilution (Figure 2c). An optimum volume would ensure the highest enrichment factor (EF) and high solvent recovery after extraction [27]. When lower volumes of chloroform below 100 μL were tested, phase separation was not observed. Hence, 100 μL of chloroform was taken as optimum for subsequent experiments.

#### 3.1.2. Type and volume of the disperser solvent

Due to their high miscibility with the extraction solvent, acetonitrile, methanol, acetone and ethanol are common disperser solvents in DLLME [23]. Since SOE was used prior to DLLME, it was decided to use acetonitrile as the disperser solvent being the most suitable solvent for SOE [28]. Volume of the disperser solvent has a significant impact on the formation of a cloudy solution, which in turn determines the extraction efficiency [23]. The effect of this parameter was investigated by using 1000 μL of the salted-out acetonitrile and adding increasing volumes of pure acetonitrile. The optimum volume of acetonitrile was found as 1250 μL (Figure 2d), beyond which peak areas decreased due to increased miscibility and solubility of chloroform and the analytes in the aqueous phase, respectively.

#### 3.1.3. Ionic strength and extraction time

In LLE, the addition of inorganic salts that increase the ionic strength has been reported to reduce the solubility of CAP in aqueous solutions [29]. However, in DLLME, such salts can also act as demulsifiers, which can eventually lead to faster phase separation, yet with reduced extraction efficiency [30]. The effect of ionic strength was evaluated by adding increasing concentrations of sodium chloride to the sample solution. It was observed that its addition caused a negative effect on the extraction efficiency (Figure 2e). Therefore, no salt was added to the sample solution in subsequent experiments. Extraction, or vortex, time corresponded to the period between the injection of the mixture of disperser/extraction solvent into the sample solution to the moment the mixture was centrifuged [19]. The effect of vortex time was monitored from 0 to 120 s. It was noticed that equilibrium was achieved within 30 s such that this value was considered optimum (Figure 2f).

### 3.2. Optimization of back-extraction conditions

Chloroform, obtained from DLLME was incompatible with reversed-phase HPLC. Hence, back-extraction of the analytes into an aqueous solution facilitated injection of the BES into the instrument. Impact of the BES volume on extraction efficiency was assessed starting from 100 to 400 μL. Extraction performance improved with an increase of up to 300 μL BES volume (Figure 2g), beyond which it started to decrease due to dilution of the analytes in excess volume of the BES. Thus, 300 μL was considered as an optimum BES volume. The effect of back-extraction time was evaluated within the range of 0 s to 60 s. Equilibrium was achieved within 15 s only due to microemulsion formation (Figure 2h). Hence, the optimum back-extraction time was taken as 15 s. 

### 3.3. Analytical performance

Both external aqueous and standard-addition calibration graphs were plotted as a function of peak area versus concentration of the three capsaicinoids under optimized conditions to test the analytical performance. External aqueous calibrations were constructed by dissolving appropriate amounts of the analytes in the BES at the concentration range of 25–150 µg mL^-1^, whereas, standard-addition calibrations were obtained by spiking the pepper samples, prior to SLE, with the standards at concentrations within the range of 2.5–15 µg mL^-1^ and applying DLLME followed by the back-extraction step. A good linearity was achieved as reflected by coefficients of determination (R^2^) in the range of 0.9962 to 0.9999 (Table 1). Intra- and interday precision, expressed as percentage relative standard deviation (%RSD), was found to be less than 6.1 and 7.5%, respectively, indicating good repeatability. Limits of detection (LOD), ranged between 0.1 and 0.3 µg mL^-1^ (or 5.0 and 15.0 µg g^-1^). Limits of quantitation (LOQ) ranged from 0.3 to 1.0 µg mL^-1^ (or 15.0 and 50.0 µg g^-1^). Linear dynamic range (LDR) extended from the LOQ to 15.0 µg mL^-1^ (0.75 mg g^-1^). The method’s EF is a measure of how sensitive this method is. The higher the EF, the more sensitive it is. EFs ranged from 3.3 to 14.7, which are significant considering the high complexity of the food samples analyzed. EFs ranged between 3.3 and 14.7 times. The proposed method’s figures merit of are summarized in Table 1.

**Table 1:** 

Method	Samplea	Analyte	Regression equationb	R^2^c	%RSDd		LODe	LOQf	LDRg	EFh
					Intraday	Interday				
ConventionalHPLC-DAD	Aqueous standards	NDHC	y=3.5(±0.1)x+1.3(±4.7)	0.9957	2.7	3.5	5.4	18.0	18.0–150	-
		CAP	y=2.1(±0.1)x-4.6(±1.5)	0.9991	1.5	2.6	2.7	9.0	9.0–150	-
		DHC	y=2.6(±0.1)x-1.5(±1.5)	0.9987	2.3	3.4	3.4	11.3	11.3–150	-
DLLME-HPLC-DAD	SGC	NDHC	y=16.9(±0.2)x-1.4(±1.1)	0.9967	5.4	7.1	0.3	1.0	1.0–15.0	4.8
		CAP	y=19.2(±0.2)x-1.6(±1.1)	0.9974	4.7	6.2	0.3	1.0	1.0–15.0	9.1
		DHC	y=18.1(±0.2)x-1.4(±1.3)	0.9962	5.8	7.5	0.3	1.0	1.0–15.0	7.0
	GPP	NDHC	y=23.2(±0.2)x-0.8(±1.1)	0.9982	3.9	4.8	0.2	0.7	0.7–15.0	6.6
		CAP	y=29.2(±0.3)x+0.4(±1.4)	0.9982	2.8	3.2	0.2	0.7	0.7–15.0	13.9
		DHC	y=38.1(±0.5)x-4.5(±2.4)	0.9973	4.9	6.3	0.3	1.0	1.0–15.0	14.7
	LGP	NDHC	y=14.0(±0.1)x-0.4(±0.3)	0.9996	1.9	2.5	0.1	0.3	0.3–15.0	4.0
		CAP	y=16.0(±0.1)x-1.0(±0.7)	0.9987	3.3	4.3	0.2	0.7	0.7–15.0	7.6
		DHC	y=22.0(±0.2)x+1.5(±1.1)	0.9979	4.1	5.4	0.2	0.7	0.7–15.0	8.5
	YP	NDHC	y=40.5(±0.3)x-1.8(±1.4)	0.9990	2.8	3.7	0.2	0.7	0.7–15.0	11.6
		CAP	y=25.1(±0.3)x-1.9(±1.8)	0.9981	4.0	5.4	0.2	0.7	0.7–15.0	12.0
		DHC	y=15.2(±0.1)x+0.9(±0.7)	0.9984	2.1	2.7	0.2	0.7	0.7–15.0	5.8
	LRP	NDHC	y=16.8(±0.2)x-2.1(±1.1)	0.9969	5.6	7.0	0.3	1.0	1.0–15.0	4.8
		CAP	y=18.7(±0.2)x-1.2(±0.8)	0.9986	2.9	3.3	0.2	0.7	0.7–15.0	8.9
		DHC	y=26.1(±0.4)x-3.5(±2.0)	0.9963	6.1	7.4	0.3	1.0	1.0–15.0	10.0
	BRP	NDHC	y=11.7(±0.1)x+0.7(±0.5)	0.9989	3.0	4.0	0.2	0.7	0.7–15.0	3.3
		CAP	y=12.8(±0.1)+0.1(±0.1)	0.9999	0.3	0.4	0.1	0.3	0.3–15.0	6.1
		DHC	y=17.6(±0.1)x-1.2(±0.8)	0.9986	3.3	4.5	0.2	0.7	0.7–15.0	6.8

b. Peak area = slope (±SD)× [analyte concentration (μg mL-1)]+ intercept (± SD).c. Coefficient of determination.d. Percentage relative standard deviation, n = 3.e. Limit of detection (µg mL^-1^).f. Limit of quantitation (µg mL^-1^).

### 3.4. Recovery studies and matrix effect 

**Figure 3:**
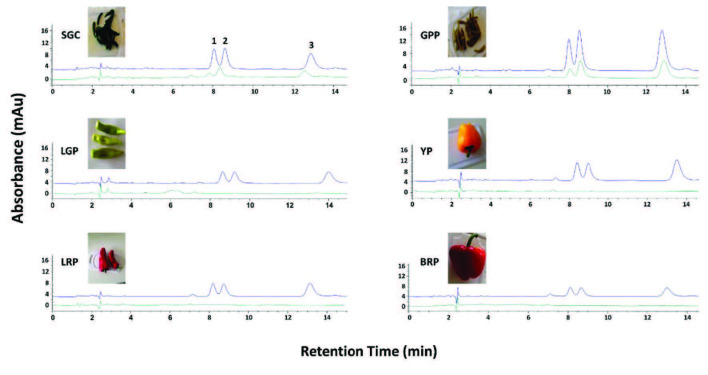
Representative chromatograms of pepper samples extracted and analyzed under optimum DLLME-HPLC-DAD conditions. Top chromatogram: sample spiked at 5.0 μg mL–1 of each analyte; bottom chromatogram: unspiked sample. Peaks: 1, NDHC; 2, CAP; and 3, DHC. SGC: small green chili; GPP: green pepper pickle; LGP: light green pepper; YP: yellow pepper; LRP: long red pepper; BRP: big red pepper.

Applicability, recovery and matrix effect were evaluated by analyzing six pepper samples for their capsaicinoid content. Typical chromatograms, shown in Figure 3, show the absence of extraneous peaks from the samples at the retention times of the three capsaicinoids, confirming high selectivity of the method. Percentage relative recoveries (%RR) obtained fro spiked samples at two concentration levels (i.e. 2.5 and 5.0 µg mL^-1^) were calculated to be in the range of 92.0–108.0 (Table 2). Possible matrix effect was assessed from the slopes of the calibration graphs for the three analytes and the samples (Table 1) and the p-value calculated using a single-factor analysis of variance (ANOVA). The difference was statistically significant (P < 0.05), suggesting the presence of matrix effect due to the wide variety of the studied samples. The standard-addition method was therefore appropriate to suppress this effect.

**Table 2:** Percentage relative recoveries of capsaicinoids from capsicum samples.

Pepper sample	Added(µg mL^-1^)	Found (µg mL^-1^)			%RRa		
		NDHC	CAP	DHC	NDHC	CAP	DHC
SGC	-	< LOQ	1.1 (±0.1)b (55 µg g–1)	< LOQ	-	-	-
	2.5	2.3 (±0.2)	2.6 (±0.1)	2.5 (±0.1)	92	104	100
	5.0	5.3 (±0.3)	5.3 (±0.3)	5.2 (±0.3)	106	106	104
GPP	-	3.2 (±0.2)(160 µg g-1)	4.8 (±0.1)(240 µg g–1)	4.4 (±0.2)(220 µg g–1)	-	-	-
	2.5	2.6 (±0.1)	2.6 (±0.1)	2.7 (±0.2)	104	104	108
	5.0	5.3 (±0.3)	5.2 (±0.2)	5.3 (±0.3)	106	104	106
LGP	-	< LOD	< LOD	< LOD	-	-	-
	2.5	2.4 (±0.1)	2.3 (±0.2)	2.7 (±0.2)	96	92	108
	5.0	5.1 (±0.1)	5.1 (±0.1)	4.9 (±0.1)	102	102	98
YP	-	< LOD	< LOD	< LOD	-	-	-
	2.5	2.3 (±0.2)	2.6 (±0.1)	2.4 (±0.1)	92	104	96
	5.0	5.1 (±0.1)	5.2 (±0.2)	4.9 (±0.1)	102	104	98
LRP	-	< LOD	< LOD	< LOD	-	-	-
	2.5	2.5 (±0.1)	2.4 (±0.1)	2.4 (±0.2)	100	96	96
	5.0	4.8 (±0.2)	4.8 (±0.3)	4.7 (±0.3)	96	96	94
BRP	-	< LOD	< LOD	< LOD	-	-	-
	2.5	2.6 (±0.1)	2.5 (±0.1)	2.4 (±0.1)	104	100	96
	5.0	4.8 (±0.2)	5.1 (±0.1)	5.0 (±0.1)	96	102	100

### 3.5. Comparison with other methods

DLLME-HPLC-DAD was contrasted with other extraction methods used to analyze capsicum samples in terms of parameters like extraction time, consumption of organic solvent used per sample, LOD, LOQ, R^2^ and %RSD. The shortest extraction time (i.e. 0.75 min) and the least volume of organic solvent (i.e. 1.9 mL) are the key benefits of this approach over the others (Table 3), with the exception of the pressurized hot-water extraction method (PHWE) suggested by Bajer et al. [31], where water was used as the extraction solvent. Nevertheless, PHWE required special instrumentation, incurring extra capital cost. Supercritical fluid extraction (SFE) proposed by Santos et al. [32] used carbon dioxide as a ‘green’ extraction solvent, however, the final extract had to be conditioned with 10 mL of methanol before injection into HPLC. The lower LODs and LOQs reported by Bajer et al. [31] and Barbero et al. [33] compared to this study were largely due to the higher sensitivity of the MS detector used in their method. MS detectors are more sensitive than UV, but they are more complicated and expensive.

**Table 3:** Comparison of DLLME-HPLC-DAD with other methods for the extraction and determination of capsaicinoids in capsicum samples.

Analytea	Extraction method/ techniqueb	Extraction time (min)	Vorg.c(mL)	LODd(µg mL^-1^)	LOQe(µg mL^-1^)	R^2^f	%RSDg	Ref.
CAP, β- carotene	SEP-HPLC	120	58	-	-	>0.9987	-	(34)
NDHC, CAP, DHC, HDHC	SFE-US-HPLC	60	10	4.10–9.76	13.67–32.55	>0.9997	-	(32)
NDHC, CAP, DHC, PAVA	PHWE-LC-APCI-MS	30	0	-	5 ng mL-1	>0.9998	-	(31)
NDHC, CAP, DHC, IDHC, HDHC	PLE-LC-MS	12	50	0.036–0.112	0.121–0.375	>0.9998	<7	(33)
NDHC, CAP, DHC	DLLME-HPLC-DAD	0.75	1.9	0.1–0.3	0.3–1.0	>0.9962	<7.5	This study

a. PAVA: nonivamide; IDHC: isodihydrocapsaicin; HDHC: homodihydrocapsaicin. b. SEP-HPLC: stage extraction process-high performance liquid chromatography; SFE-US-HPLC: supercritical fluid extraction- ultrasound-high performance liquid chromatography; PHWE-LC-APCI-MS: pressurized hot-water extraction-liquid chromatography-atmospheric pressure chemical ionization-mass spectrometry; PLE-LC-MS: pressurized liquid extraction-liquid chromatography-mass spectrometry. c. Total volume of organic extraction solvent used. d. Limit of detection (µg mL^-1^ f. Coefficient of determination.g. Percentage relative standard deviation, n = 3.

## 4. Conclusion

DLLME, combined with a back-extraction step, was proposed prior to HPLC-DAD for the extraction of the three major capsaicinoids from different cultivars of pepper (*Capsicum annuum* L.) samples. Several advantages of this method as compared to other existing extraction methods include cost-effectiveness, rapidness, high extraction efficiency, good selectivity and sample clean up as well as minimum consumption of organic solvents. Despite the complexity of the studied matrix, high recoveries, good linearity, reproducibility and interference-free chromatograms were attained. These advantages of DLLME-HPLC-DAD encourages its use for the quantitation of capsaicinoids in pepper samples in routine food analysis and quality control laboratories. The ability to scale-up DLLME for the isolation of capsaicinoids with high purity suggests that this method be applied for the isolation of various intermediate- to low-polarity compounds from natural products, which reduces the extraction time and organic solvent consumption, satisfying the criteria for green chemistry.

## References

[ref1] (2017). Contents of capsaicinoids in chillies grown in Denmark. Food Chemisty.

[ref2] (2017). Extraction, bioavailability, and bioefficacy of capsaicinoids. Journal of Food and Drug Analysis.

[ref3] (2009). Analysis of capsaicin and dihydrocapsaicin in peppers and pepper sauces by solid phase microextraction-gas chromatography-mass spectrometry. Journal of Chromatography A.

[ref4] (2014). Bioavailability of capsaicin and its implications for drug delivery. Journal of Controlled Release.

[ref5] (2014). Effect of sowing time and crop geometry on the Capsaicinoid content in Bhoot Jolokia (Capsicum chinense Jacq. Journal of Food Science and Technology-Mysore.

[ref6] (2016). Capsaicin suppresses cell proliferation, induces cell cycle arrest and ROS production in bladder cancer cells through FOXO3a-mediated pathways. Molecules.

[ref7] (2013). Role of the capsaicin-sensitive sensory nerves in autoantibody-induced arthritis of the mouse. Journal of Molecular Neuroscience.

[ref8] (2014). Capsaicin as therapeutic molecule. In: Rainsford KD (editor).

[ref9] (2006). Analysis of the contents of pungent compounds in fresh Korean red peppers and in pepper-containing foods. Journal of Agricultural and Food Chemistry.

[ref10] (2012). A comprehensive review of the carcinogenic and anticarcinogenic potential of capsaicin. Toxicologic Pathology.

[ref11] (2002). Ground red peppers: capsaicinoids content, Scoville scores, and discrimination by an electronic nose. Journal of Agricultural and Food Chemistry.

[ref12] (2004). Practical aspects of fast HPLC separations for pharmaceutical process development using monolithic columns. Analytica Chimica Acta.

[ref13] (1997). Characterization and quantitation of capsaicins in natural and commercial products by HPLC-UV and electrospray LC-MS. Abstracts of Papers of the American Chemical Society.

[ref14] (2012). Techawongstien S. Determination of capsaicin and dihydrocapsaicin in some chilli varieties using accelerated solvent extraction associated with solid-phase extraction methods and RP-HPLC-fluorescence. E-Journal of Chemistry.

[ref15] (1985). Formation and metabolism of pungent principle of capsicum fruits: XV. microdetermination of capsaicin by high-performance liquid-chromatography with electrochemical detection. Journal of Chromatography.

[ref16] (2007). Inheritance of capsaicin and dihydrocapsaicin, determined by HPLC-ESI/MS, in an intraspecific cross of Capsicum annuum L. Journal of Agricultural and Food Chemistry.

[ref17] (2004). Comparison of performance of C18 monolithic rod columns and conventional C18 particle-packed columns in liquid chromatographic determination of estrogel and ketoprofen gel. Journal of Chromatography B-Analytical Technologies in the Biomedical and Life Sciences.

[ref18] (2011). Dispersive liquid-liquid microextraction. Trac-Trends in Analytical Chemistry.

[ref19] (2006). Determination of organic compounds in water using dispersive liquid-liquid microextraction. Journal of Chromatography A.

[ref20] (2013). Dispersive liquid-liquid microextraction combined with field-amplified sample stacking in capillary electrophoresis for the determination of non-steroidal anti-inflammatory drugs in milk and dairy products. Food Chemistry.

[ref21] (2014). -liquid microextraction in food analysis. A critical review. Analytical and Bioanalytical Chemistry.

[ref22] (2015). Determination of parabens in human milk and other food samples by capillary electrophoresis after dispersive liquid–liquid microextraction with back-extraction. Food Chemistry.

[ref23] (2017). Ten years of dispersive liquid-liquid microextraction and derived techniques. Applied Spectroscopy Reviews.

[ref24] (2020). Switchable-hydrophilicity solvent liquid-liquid microextraction versus dispersive liquid-liquid microextraction prior to HPLC-UV for the determination and isolation of piperine from Piper nigrum L. Journal of Separation Science.

[ref25] (2010). Evolution of dispersive liquid-liquid microextraction method. Journal of Chromatography A.

[ref26] (2008). -liquid microextraction method based on solidification of floating organic drop combined with gas chromatography with electron-capture or mass spectrometry detection. Journal of Chromatography A.

[ref27] (2010). Dispersive liquid-liquid microextraction based on the solidification of floating organic drop followed by inductively coupled plasma-optical emission spectrometry as a fast technique for the simultaneous determination of heavy metals. Journal of Chromatography A.

[ref28] (2013). Salting-out assisted liquid-liquid extraction for bioanalysis. Bioanalysis.

[ref29] (2017). Extraction and purification of capsaicin from capsicum oleoresin using an aqueous two-phase system combined with chromatography. Journal of Chromatography B-Analytical Technologies in the Biomedical and Life Sciences.

[ref30] (2015). Vortex-assisted low density solvent liquid-liquid microextraction and salt-induced demulsification coupled to high performance liquid chromatography for the determination of five organophosphorus pesticide residues in fruits. Talanta.

[ref31] (2015). Central composite design of pressurised hot water extraction process for extracting capsaicinoids from chili peppers. Journal of Food Composition and Analysis.

[ref32] (2015). Supercritical carbon dioxide extraction of capsaicinoids from malagueta pepper (Capsicum frutescens L.) assisted by ultrasound. Ultrasonics Sonochemistry.

[ref33] (2006). Pressurized liquid extraction of capsaicinoids from peppers. Journal of Agricultural and Food Chemistry.

[ref34] (2014). Stage extraction of capsaicinoids and red pigments from fresh red pepper (Capsicum) fruits with ethanol as solvent. Lwt-Food Science and Technology.

